# Arbitrary entanglement of three qubits via linear optics

**DOI:** 10.1038/s41598-022-22835-4

**Published:** 2022-12-14

**Authors:** Pawel Blasiak, Ewa Borsuk, Marcin Markiewicz

**Affiliations:** 1 Institute for Quantum Studies, Chapman University, Orange, CA 92866 USA; 2 Institute of Nuclear Physics Polish Academy of Sciences, 31342 Kraków, Poland; 3 International Centre for Theory of Quantum Technologies, University of Gdańsk, 80308 Gdańsk, Poland

**Keywords:** Quantum physics, Quantum information, Qubits, Single photons and quantum effects, Optical physics

## Abstract

We present a linear-optical scheme for generating an arbitrary state of three qubits. It requires only three independent particles in the input and post-selection of the coincidence type at the output. The success probability of the protocol is equal for any desired state. Furthermore, the optical design remains insensitive to particle statistics (bosons, fermions or anyons). This approach builds upon the no-touching paradigm, which demonstrates the utility of particle indistinguishability as a resource of entanglement for practical applications.

Entanglement remains a central theme in quantum foundations research^1,2^. It is considered a key resource enabling the advantage in quantum information tasks^3,4^. There is therefore a vital interest in practical entanglement generation schemes capable of delivering the broadest possible range of states, from which one might choose a state desired for a problem at hand. Ideally, we would like to have a generic and efficient tool for constructing an arbitrary multi-particle state from some simpler initial state (possibly having just a few independent particles to begin with). A paradigmatic example is the construction of the full class of single-particle states (a qudit). In this case, arbitrary unitary transformation can be experimentally implemented by linear-optical devices^5^ and hence any single-particle state can be deterministically prepared from any given initial state. However, it is not true for multi-particle states that do not transform one into another by means of linear optics^6^. Thus for the generation of multi-partite entanglement, non-linear effects or post-selection of some sort must be employed. Several techniques have been developed to this effect which produce certain classes of states^7–10^. However, there is no systematic linear-optical method for obtaining an arbitrary multi-particle entangled state that would start with a supply of independent particles in the input.

In this work, we focus on entanglement generation for three qubits. Notably, this is the first non-trivial case where the arbitrariness of the desired state becomes challenging. An interesting approach to this problem consists in considering the set of stochastic local operations and classical communication (SLOCC)^11–13^. It has been shown that for three qubits there are two inequivalent classes of genuinely tripartite entangled states^12^. This means that using SLOCCs for filtering arbitrary tripartite entanglement requires two different types of entangled states to begin with, i.e., states of the GHZ and the W type. However, it should be remarked that the efficiency of such protocols drops to zero when moving away from the initial state. Another important result concerns the full set of linear optical transformations (without post-selection)^6^. Then the situation is further complicated as for three qubits the set of entangled states splits into a continuous number of inequivalent classes. Those results illustrate the difficulties in efficiently generating arbitrary entanglement using linear operations without any entanglement from the outset.

Here we will show that linear-optical transformations augmented with post-selection of the coincidence type are enough to generate arbitrary entanglement of three qubits from three independent particles (i.e., without requiring any prior entanglement). Our proposal builds on the so-called no-touching paradigm in optical designs which draws from the inherent indistinguishability of particles; see Ref.^14^ for a general scheme and Refs.^15–19^ for some examples. Notably, the protocol is an instance of direct and explicit construction of any given state. A distinctive advantage of the proposal is that it has equal efficiency for generating any desired state and it is insensitive to particle statistics.

**Figure 1:**
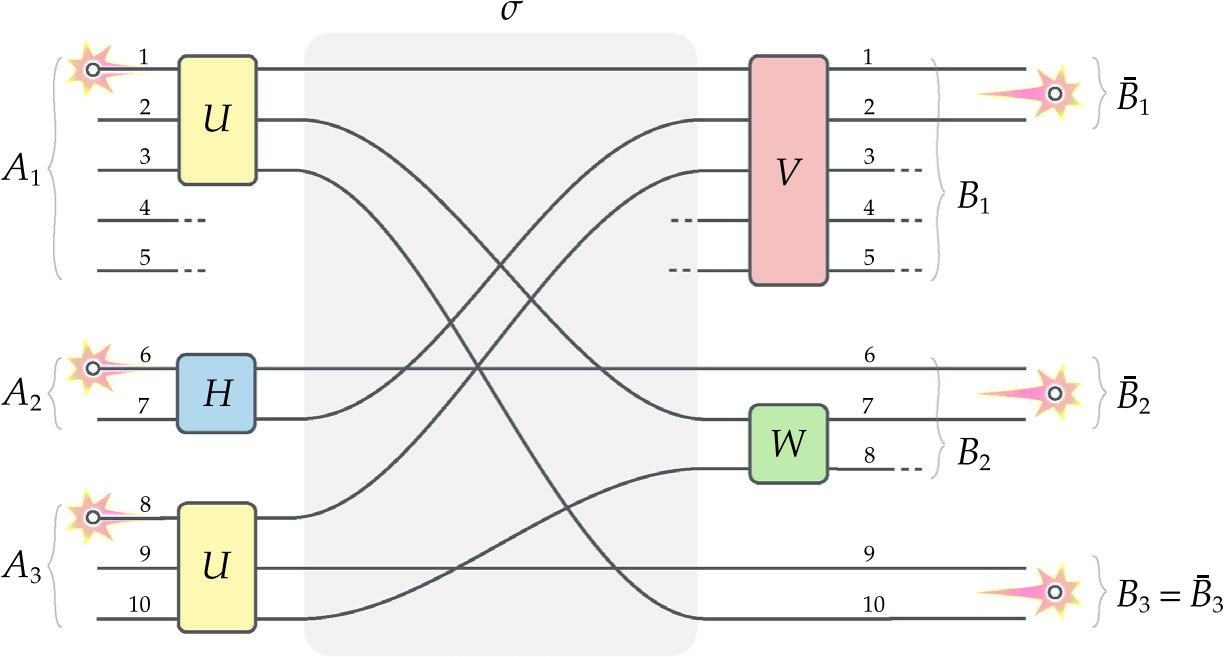
**No-touching design for arbitrary state of three qubits.** Three independent identical particles entering the optical circuit undergo a sequence of transformations which consists of local unitaries *U*, *H* and *U* on subsystems $A_1$, $A_2$ and $A_3$, followed by permutation of the paths $\sigma$, and again local unitaries *V* and *W* on subsystems $B_1$ and $B_2$. Post-selection on a single particle in each of the dual-rail qubits generates an arbitrary state of three qubits $\bar{B}_1$, $\bar{B}_2$ and $\bar{B}_3$ in Eq. (2) by the appropriate choice of unitaries in Eq. (6) as specified in Eqs. (18)–(20).

## Results

### Simplification by generalised Schmidt decomposition

A general state of three qubits reads

1 $$\begin{aligned} \mathinner {|{\psi }\rangle }= & {} \sum _{ijk=0,1}\alpha _{ijk}\mathinner {|{ijk}\rangle }, \end{aligned}$$

where $\mathinner {|{ijk}\rangle }$ is a computational basis in $\mathbb {C}^2\otimes \mathbb {C}^2\otimes \mathbb {C}^2$. It turns out that this can be simplified by local transformations to the combination of the following five states

2 $$\begin{aligned} \mathinner {|{\psi }\rangle }= & \ {} a\mathinner {|{000}\rangle }+b\,e^{i\varphi }\mathinner {|{100}\rangle }+c\mathinner {|{110}\rangle }+d\mathinner {|{101}\rangle }+e\mathinner {|{111}\rangle }, \end{aligned}$$

with five real parameters $a,b,c,d,e\ge 0$ and one phase $0\le \varphi \le \pi$, such that

3 $$\begin{aligned} a^2+b^2+c^2+d^2+e^2=\ & {} 1. \end{aligned}$$

This follows from the generalised Schmidt decomposition for three qubits^20,21^ and no further reduction of the number of non-vanishing terms is possible.

Therefore, to generate the state in Eq. (1), it is enough to provide the state in Eq. (2) and then make a local transformation on each qubit. In the following, we give the explicit protocol for optical construction of an arbitrary state in the reduced form of Eq. (2).

### Optical realisation

Consider three identical particles injected into an optical circuit which consists of 10 paths (or modes). The particle statistics (bosons, fermions or anyons) is irrelevant for the purpose at hand. Following the idea presented in Ref.^14^ we will group paths at the input and output into three systems, see Fig. 1:

$$\begin{aligned} \!\!\!\!\!\!&\textit{Input:}\ \ A_1=\{1,2,3,4,5\},\ A_2=\{6,7\},\ A_3=\{8,9,10\},\\ \!\!\!\!\!\!&\textit{Output:}\ \ B_1=\{1,2,3,4,5\},\ B_2=\{6,7,8\},\ B_3=\{9,10\}. \end{aligned}$$

Furthermore, we will distinguish three pairs of paths $\bar{B}_1$, $\bar{B}_2$ and $\bar{B}_3$ in the respective subsystems at the output, i.e. $\bar{B}_k\subset B_k$. They will play the role of the so-called *dual-rail qubits*, where the computational basis $\mathinner {|{0}\rangle }$,$\mathinner {|{1}\rangle }$ is encoded by the presence of a single particle in the respective path of a given pair $\bar{B}_k$. Accordingly, we have the following representation for a general qubit state $\alpha \mathinner {|{0}\rangle }+\beta \mathinner {|{1}\rangle }$:

$$\begin{aligned} \begin{array}{lcl} \bar{B}_1=\{1,2\}&{} \leadsto &{}\textit{Qubit 1: }(\alpha \, a^\dag _1+\beta \, a^\dag _2)\mathinner {|{\Omega }\rangle },\\ \bar{B}_2=\{6,7\}&{} \leadsto &{}\textit{Qubit 2: }(\alpha \, a^\dag _6+\beta \, a^\dag _7)\mathinner {|{\Omega }\rangle },\\ \bar{B}_3=\{9,10\}&{} \leadsto &{}\textit{Qubit 3: }(\alpha \, a^\dag _9+\beta \, a^\dag _{10})\mathinner {|{\Omega }\rangle }. \end{array} \end{aligned}$$

where $\mathinner {|{\Omega }\rangle }$ denotes the vacuum state and $a^\dag _i$ are the usual particle creation operators in the second quantisation formalism. We note that the dual-rail encoding assumes the presence of a *single* particle in a given pair of paths $\bar{B}_k$ for $k=1,2,3$. In our scheme this will be guaranteed by *post-selection* on a single particle in each dual-rail qubit $\bar{B}_k$.

The optical protocol is depicted in Fig. 1. It consists of a sequence of three unitary transformations on three independent particles injected in paths 1, 6 and 8. First, the particles undergo local unitaries *U*, *H* and *U* in each subsystem $A_1$, $A_2$ and $A_3$. Second, the paths are rearranged according to some permutation $\sigma \in \mathcal {S}_{10}$. Third, there are two local unitaries *V* and *W* on subsystems $B_1$ and $B_2$ implemented at the output. Finally, the protocol ends with post-selection on a single particle in each pair of paths $\bar{B}_1$, $\bar{B}_2$ and $\bar{B}_3$ which generates three dual-rail encoded qubits.

Now, we can make the unitaries in Fig. 1 more precise. Let the first two transformations *U* and *V* produce symmetric superposition of the injected particles, which in the matrix notation amounts to

4 $$\begin{aligned} U\,=\, \tfrac{1}{\sqrt{3}}\left( \begin{array}{ccc} 1&{}{\textbf {.}}&{}{\textbf {.}}\\ 1&{}{\textbf {.}}&{}{\textbf {.}}\\ 1&{}{\textbf {.}}&{}{\textbf {.}}\\ \end{array} \right) \qquad { \text{and} }\qquad H\, =\, \tfrac{1}{\sqrt{2}}\left( \begin{array}{rr} 1&{}{\textbf {.}}\\ 1&{}{\textbf {.}} \end{array} \right) . \end{aligned}$$

The permutation of modes $\sigma \in \mathcal {S}_{10}$ is given as follows

5 $$\begin{aligned} \sigma= \ {} \left( \begin{array}{cccccccccc} 1&{}2&{}3&{}4&{}5&{}6&{}7&{}8&{}9&{}10\\ 1&{}7&{}10&{}4&{}5&{}6&{}2&{}3&{}9&{}8 \end{array} \right) . \end{aligned}$$

The final unitaries *V* and *W* are defined in a non-trivial way by the following two matrices

6 $$\begin{aligned} V\,=\, \left( \begin{array}{ccccc} \kappa &{}0&{}0&{}\delta &{}\epsilon \\ \bar{\delta }&{}\mu &{}\nu &{} \text{-}\,\bar{\kappa }&{}0\\ {\textbf {.}}&{}{\textbf {.}}&{}{\textbf {.}}&{}{\textbf {.}}&{}{\textbf {.}}\\ {\textbf {.}}&{}{\textbf {.}}&{}{\textbf {.}}&{}{\textbf {.}}&{}{\textbf {.}}\\ {\textbf {.}}&{}{\textbf {.}}&{}{\textbf {.}}&{}{\textbf {.}}&{}{\textbf {.}} \end{array} \right) \qquad \text{and} \qquad W\,=\, \left( \begin{array}{cc} \xi &{}\tau \\ {\textbf {.}}&{}{\textbf {.}} \end{array} \right) , \end{aligned}$$

with some parameters $\kappa ,\delta ,\nu ,\mu ,\epsilon ,\xi$ and $\tau$. In the above notation, the dots “${\textbf {.}}$” are left unspecified and chosen so that the matrices are unitary. Observe that this can always be done by augmenting the missing columns/rows to an orthonormal basis (note that the two upper rows of *V* are orthogonal at the outset). The only constraint on the parameters $\kappa ,\delta ,\nu ,\mu ,\epsilon ,\xi$ and $\tau$ is their respective normalisation, i.e.

7 $$\begin{aligned} |\xi |^2+|\tau |^2= \ {} 1, \end{aligned}$$

8 $$\begin{aligned} |\kappa |^2+|\delta |^2+|\epsilon |^2= \ {} 1, \end{aligned}$$

9 $$\begin{aligned} |\delta |^2+|\mu |^2+|\nu |^2+|\kappa |^2= \ {} 1. \end{aligned}$$

For our purposes, the dotted entries “${\textbf {.}}$” will play no role in the argument (in the following, they contribute only to the terms that drop out upon post-selection). All the remaining parameters $\kappa ,\delta ,\nu ,\mu ,\epsilon ,\xi$ and $\tau$ will be specified shortly.

Let us write out the state that results from the protocol in Fig. 1 after injecting three independent particles in paths 1, 6, 8 and post-selecting on a single particle in each dual-rail qubit $\bar{B}_1$, $\bar{B}_2$ and $\bar{B}_3$. The evolution of the system is given by a sequence of steps as described in the following lines:

10 $$ a_{1}^{\dag } a_{6}^{\dag } a_{8}^{\dag } \left| \Omega \right\rangle \xrightarrow[{Eq.(4)}]{{U,H,U}}\tfrac{1}{{3\sqrt 2 }}\big (\,a_{1}^{\dag }  + a_{2}^{\dag }  + a_{3}^{\dag } \,\big )\big (\,a_{6}^{\dag }  + a_{7}^{\dag } \,\big )\big (\,a_{8}^{\dag }  + a_{9}^{\dag }  + a_{{10}}^{\dag } \,\big )\left| \Omega \right\rangle  $$

11 $$ \begin{aligned} \qquad \xrightarrow[{Eq.(5)}]{\sigma }\tfrac{1}{{3\sqrt 2 }}\big (\,a_{1}^{\dag }  + a_{7}^{\dag }  + a_{{10}}^{\dag } \,\big )\big (\,a_{6}^{\dag }  + a_{2}^{\dag } \,\big )\big (\,a_{3}^{\dag }  + a_{9}^{\dag }  + a_{8}^{\dag } \,\big )\left| \Omega \right\rangle  \end{aligned} $$

12 $$ \begin{aligned} \qquad \xrightarrow[{Eq.(6)}]{{V,W}}\tfrac{1}{{3\sqrt 2 }}\Big ( {\big(\kappa a_{1}^{\dag }  + \bar{\delta }a_{2}^{\dag }  + ...\big) + \big(\xi a_{7}^{\dag }  + ...\big) + a_{{10}}^{\dag } } \Big ) \end{aligned} $$

13 $$\begin{aligned}&\qquad \qquad \!\Big (\,a^{\dag }_{\scriptscriptstyle 6}+\big (\,\mu \,a^{\dag }_{\scriptscriptstyle 2}+ \cdot\cdot\cdot \,\big )\Big )\, \Big (\big (\,\nu \,a^{\dag }_{\scriptscriptstyle 2}+ \cdot\cdot\cdot \,\big )+a^{\dag }_{\scriptscriptstyle 9}+\big (\,\tau \,a^{\dag }_{\scriptscriptstyle 7}+\cdot\cdot\cdot \,\big )\Big )\mathinner {|{\Omega }\rangle }\end{aligned}$$

14 $$\begin{aligned} \qquad  \mathop{ \rightsquigarrow }\limits^{{post{\text{-}}select}}  \tfrac{1}{{3\sqrt 2 }}(\kappa a_{1}^{\dag } a_{6}^{\dag } a_{9}^{\dag }  + \bar{\delta }a_{2}^{\dag } a_{6}^{\dag } a_{9}^{\dag }  + \xi \mu a_{7}^{\dag } a_{2}^{\dag } a_{9}^{\dag }  + \nu a_{{10}}^{\dag } a_{6}^{\dag } a_{2}^{\dag }  + \mu \tau a_{{10}}^{\dag } a_{2}^{\dag } a_{7}^{\dag } )\left| \Omega \right\rangle  \end{aligned}$$

15 $$\begin{aligned}& \qquad  \qquad  =\ \tfrac{1}{3\sqrt{2}}\,\Big (\,\kappa \mathinner {|{000}\rangle }+\bar{\delta }\mathinner {|{100}\rangle }+\xi \mu \mathinner {|{110}\rangle }+\nu \mathinner {|{101}\rangle }+\mu \tau \mathinner {|{111}\rangle }\Big ), \end{aligned}$$

where the last equality holds for bosons in the dual-rail encoding. This renders the desired state in Eq. (2) when

16 $$\begin{aligned} \kappa =a,\ \ \bar{\delta }=b\,e^{i\varphi },\ \ \xi \mu =c,\ \ \nu =d,\ \ \mu \tau =e. \end{aligned}$$

We observe that we can always choose matrices *W* and *V* to satisfy these equations by defining parameters $\kappa ,\delta ,\nu ,\mu ,\epsilon ,\xi$ and $\tau$ in the following way

17 $$\begin{aligned}&\kappa =a,\qquad \delta =b\,e^{-i\varphi },\qquad \nu =d, \end{aligned}$$

18 $$\begin{aligned}{}&\quad \mu =\sqrt{c^2+e^2},\qquad \epsilon =\sqrt{1-a^2-b^2}, \end{aligned}$$

19 $$\begin{aligned}&\quad \xi =\frac {\textstyle {c}}{\textstyle {\mu }}\,,\qquad \tau =\frac {\textstyle {e}}{\textstyle {\mu }}.\ \ \end{aligned}$$

(If $\mu =0$, then $\xi$ and $\tau$ can be taken arbitrarily). It is straightforward to check that the conditions in Eqs. (7)–(9) are satisfied, since the constraint in Eq. (3) holds.

Note that the scheme works for fermions as well. In this case, the final state in Eq. (16) takes the form

20 $$\begin{aligned} \!\!\!\!\!\!\!\!\!\kappa \mathinner {|{000}\rangle }+\bar{\delta }\mathinner {|{100}\rangle }-\xi \mu \mathinner {|{110}\rangle }-\nu \mathinner {|{101}\rangle }+\mu \tau \mathinner {|{111}\rangle }, \end{aligned}$$

and then the Eqs. (18)–(20) require a trivial modification $\nu =-\,d$ and $\xi =-\,\frac {\textstyle {c}}{\textstyle {\mu }}$ in order to recover the desired state in Eq. (2). In a similar manner it is straightforward to adjust Eq. (16) for *any* particle statistics (anyons).

Finally, we observe that the expression in Eq. (16) is unnormalised due to post-selection. This allows to read off the success probability (efficiency) of the process which is equal to $\big (\,\frac {1}{3\sqrt{2}}\,\big )^{\scriptscriptstyle 2}=\frac {1}{18}\approx 5\%$. Notably, the efficiency is the *same* for *every* three-qubit state $\mathinner {|{\psi }\rangle }$.

## Discussion

We remark that the above-described protocol, based on dual-rail encoded qubits, provides a ready-made template that straightforwardly translates into any other physical implementation of qubits. This turns out to be a generic feature of the no-touching designs in which the question of particle statistics becomes virtually irrelevant because of post-selection. It is due to the fact that the latter projects on the sector where the evolution of the system features at most a single particle in each mode, which makes immaterial the distinction between the bunching and anti-bunching effects for bosons and fermions; see Refs.^14–19^ for a discussion.

From the fundamental point of view, it is interesting to note the significance of the inherent indistinguishability of particles as conveniently described in the second quantisation formalism. It appears that entanglement resulting from the symmetrization postulate can be treated as a genuine resource and transformed into other kinds of entanglement which can be directly observed and used for practical applications^14,22–24^. This paper shows that arbitrary entanglement of three qubits can be extracted in this way. For an extension to some multi-particle entangled states see e.g. Refs.^19,25–29^.

An important advantage of the proposed protocol is the minimal amount of resources employed to generate an arbitrary three-qubit state compared to the existing techniques:

- It requires *only* linear optics and works equally well for *any* particle statistics, cf. Refs.^7–10^.
- There is *no* need for auxiliary systems (particles) or measurements, cf. Refs.^25,27^.
- The protocol requires *only* three independent particles in the input, i.e., no prior entanglement is required.
- It has the *same* efficiency for the generation of *any* desired three-qubit state.

This distinguishes our proposal from the typical approach based on filtering via SLOCC operations which requires auxiliary entanglement from the outset, and its success probability for arbitrary three-qubit states drops to zero; see **Methods** section for discussion. Moreover, the generation of states via SLOCC filtering generally demands different initial states depending on the SLOCC equivalence class of the target state. For optical proposals aimed at preparation of single representatives in the SLOCC classes for the purpose of filtering see Refs.^14,26,28,30^. Notably, our protocol overcomes the division into SLOCC equivalence classes due to the presence of mode permutation $\sigma$, which is a non-local operation from the point of view of subsystems defined by mode grouping.

We note that our scheme relies on a specific type of post-selection which requires *coincidence* count in the output channels encoding dual-rail qubits $\bar{B}_1$, $\bar{B}_2$ and $\bar{B}_3$. A direct way to check the coincidence criterion involves the measurement of each qubit. Typically this destroys the state, but the recorded correlations can be still used for the extraction of some information relevant to a given experiment. The utility of such a direct post-selection depends on the task at hand. For illustration see, e.g., the recent boson sampling experiments^31^ or the direct verification of the boson nature of photons^32,33^. It was also shown to be safe for Bell-type tests of non-locality^24^. We remark that many modern state generation schemes refer to post-selection of the the coincidence-type, like e.g. entanglement by path identity^9,34,35^ or spatial overlap of indistinguishable particles^22,23,36^. However, if the generated state needs to be further processed, then direct detection does not meet this requirement. In such a case, the solution is provided by non-demolition measurements carried out on each dual-rail qubit $\bar{B}_1$, $\bar{B}_2$ and $\bar{B}_3$. Such a measurement ascertains the presence of a particle without destroying it and not affecting its state. Therefore, a positive joint result of those three (non-demolition) measurements heralds the generation of the desired state in the signal modes. This turns the protocol into an *event-ready* scheme. Notably, a few non-demolition measurement techniques have been developed in recent years. See e.g. Refs.^37–39^ for non-demolition detection of photons (noting that polarization and dual-rail encoding of qubits transforms one into another via polarizing beam splitters).

In summary, the characteristic features of the proposed scheme for state generation are marked by simplicity (just linear optics and post-selection of the coincidence type), limited initial resources (just three independent particles in the input), and universal efficiency (equal for any desired state). This makes the proposal an interesting technique for integrated quantum technologies motivating further research towards an extension to an arbitrary number of qubits; see the recent progress in the optical generation of certain multi-particle states within the no-touching paradigm^19,25–28,30^. We also indicate possible further improvements using a graph-theoretical approach to the analysis of linear optical schemes^40^.

## Methods

### Comparison with the generation of arbitrary states via SLOCC operations

In this paper, we presented a universal interferometric protocol for generating an arbitrary three-qubit state from an input product state of three particles, which prepares an arbitrary state with constant finite efficiency. Here, we compare this scheme with the generation of arbitrary states from a GHZ class starting from the GHZ input state via SLOCC operations. We will see that the lattr method of state generation has a vanishing efficiency for some states in this class.

As shown in the seminal paper by Dür et al.^12^ an arbitrary state from the GHZ class can be parametrised by five real parameters as

21 $$\begin{aligned}{}&\mathinner {|{\psi _{\text {GHZ}}(\chi ,\theta ,\alpha _1,\alpha _2,\alpha _3)}\rangle }=\ \sqrt{K}\left( \cos (\chi )\mathinner {|{000}\rangle }+\sin (\chi )e^{i\theta }\mathinner {|{s_1}\rangle }\mathinner {|{s_2}\rangle }\mathinner {|{s_3}\rangle }\right) , \end{aligned}$$

in which the normalisation constant reads $K=(1+2\cos (\chi )\sin (\chi )\cos (\alpha _1)\cos (\alpha _2)\cos (\alpha _3)\cos (\theta ))^{-1}$ and the states $\mathinner {|{s_i}\rangle }$ are given by $\cos (\alpha _i)\mathinner {|{0}\rangle }+\sin (\alpha _i)\mathinner {|{1}\rangle }$. The ranges of the parameters are as follows: $\chi \in (0,\frac{\pi }{4}]$, $\alpha _i\in (0, \frac{\pi }{2}]$ and $\theta \in [0,2\pi )$. This state can be obtained from the standard GHZ state $\mathinner {|{\psi _{\text {GHZ}}}\rangle }=\frac{1}{\sqrt{2}}(\mathinner {|{000}\rangle }+\mathinner {|{111}\rangle })$ via SLOCC filtering operations specified by

22 $$\begin{aligned} \mathinner {|{\psi _{\text {GHZ}}(\chi ,\theta ,\alpha _1,\alpha _2,\alpha _3)}\rangle }=M(\chi ,\theta ,\alpha _1,\alpha _2,\alpha _3)\mathinner {|{\psi _{\text {GHZ}}}\rangle }, \end{aligned}$$

where the SLOCC operator *M* has the form^12^

23 $$\begin{aligned} M(\chi ,\theta ,\alpha _1,\alpha _2,\alpha _3)= &\ {} \sqrt{2K}\left( \begin{array}{ll} \cos (\chi )&{}\sin (\chi )\cos (\alpha _1)e^{i\theta }\\ 0&{}\sin (\chi )\sin (\alpha _1)e^{i\theta } \end{array} \right) \otimes \left( \begin{array}{ll} 1&{}\cos (\alpha _2)\\ 0&{}\sin (\alpha _2) \end{array} \right) \otimes \left( \begin{array}{ll} 1&{}\cos (\alpha _3)\\ 0&{}\sin (\alpha _3) \end{array} \right) \nonumber \\= &\ {} \sqrt{2K}\,\tilde{M}(\chi ,\theta ,\alpha _1,\alpha _2,\alpha _3). \end{aligned}$$

Such a filtering operation can be implemented as a two-outcome POVM measurement^13^, with measurement operators defined by $P=M/||M||$ and $P'=\sqrt{\mathbbm{1}-P^{\dagger }P}$. The outcome related to the measurement operator *P* indicates the success of the protocol, whereas the outcome related to $P'$—its failure. The norm has to be chosen to guarantee that $P^{\dagger }P\le \mathbbm{1}$. One of the typical choices is the spectral norm of the operator *M*, defined as the largest singular value of *M*. This choice turns out to be optimal for the task of entanglement distillation of two-qubit states^13^. However, other choices that guarantee the condition $P^{\dagger }P\le \mathbbm{1}$, such as the Frobenius norm, are also correct. The success probability of filtering arbitrary state of the form Eq. (22) for SLOCC operator *M* is thus given by^41^

24 $$\begin{aligned} p_{\text {succ}}\ = \ & {} {\text {Tr}}(P\rho _{\text {GHZ}}P^{\dagger })\ =\ {\text {Tr}}\left( \frac{M}{||M||}\rho _{\text {GHZ}}\frac{M^{\dagger }}{||M^{\dagger }||}\right) \ = {}\ \frac{{\text {Tr}}(M\rho _{\text {GHZ}}M^{\dagger })}{||M||^2}\ =\ \frac{1}{||M||^2}, \end{aligned}$$

where $\rho _{\text {GHZ}}=\mathinner {|{\psi _{\text {GHZ}}}\rangle }\mathinner {\langle {\psi _{\text {GHZ}}}|}$, and the last equality follows from the fact that $\mathinner {|{\psi _{\text {GHZ}}}\rangle }$ is already properly normalised. Note that the operator *M* is *not* unitary, and therefore it does not preserve the normalisation of a general state it acts on—the state $\rho _{\text {GHZ}}$ is an exception.

**Figure 2:**
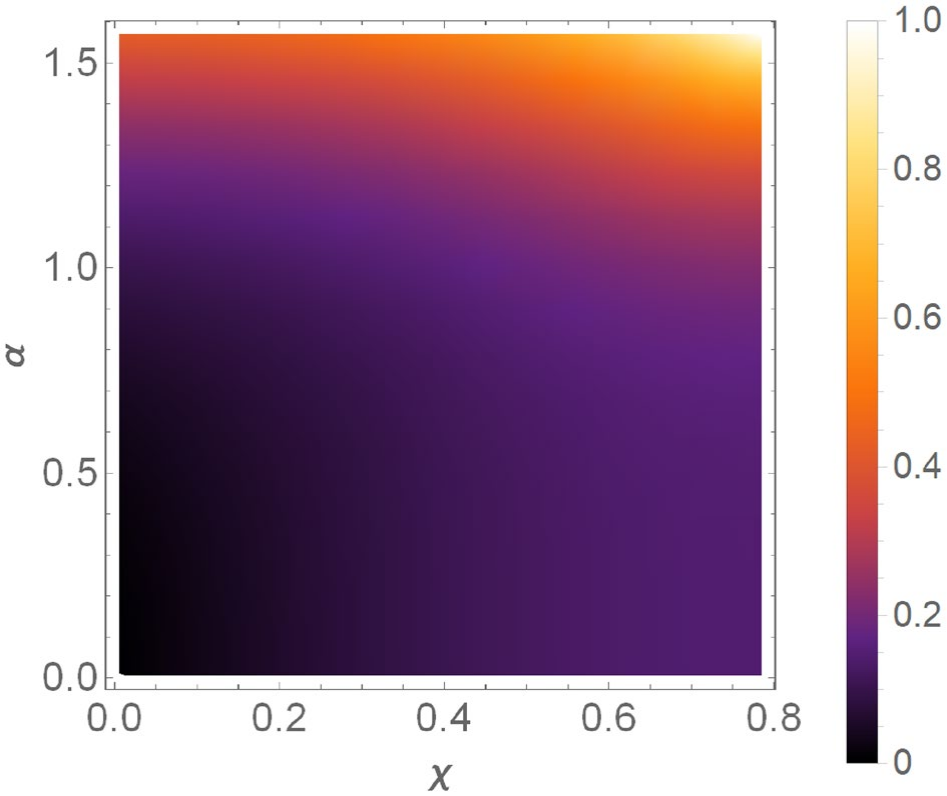
Success probability of obtaining arbitrary state from a two-parameter subclass of the GHZ class in Eq. (22) of the form $\mathinner {|{\psi _{\text {GHZ}}(\chi ,\pi ,\alpha ,\alpha ,\alpha )}\rangle }$. For $\chi =\frac{\pi }{4}$ and $\alpha \rightarrow 0$, the success probability vanishes, indicating that these states cannot be effectively obtained via SLOCC filtering.

Let us assume that we choose the spectral norm in Eq. (25). For the clarity of presentation we will focus on a two-parameter subclass of states from the GHZ class Eq. (22) of the form $\mathinner {|{\psi _{\text {GHZ}}(\chi ,\pi ,\alpha ,\alpha ,\alpha )}\rangle }$. In Fig. 2 we present the success probability of obtaining this state from the GHZ state as a function of parameters $\chi$ and $\alpha$. We can see that the probability tends to zero for $\chi =\frac{\pi }{4}$ and $\alpha \rightarrow 0$, which stands in sharp contrast with our protocol that allows for the generation of these states with the fixed finite probability of success independently of the values of the parameters. One may argue that the effect of vanishing probability is related to a specific choice of the norm. However, it is easy to see that this effect holds for any choice of the norm consistent with the condition $P^{\dagger }P\le \mathbbm{1}$. Indeed, it suffices to show that $||M(\tfrac{\pi }{4},\pi ,\alpha \rightarrow 0)||\rightarrow \infty$ for any choice of the norm. Due to Eq. (24) we have

25 $$\begin{aligned}&||M(\tfrac{\pi }{4},\pi ,\alpha \rightarrow 0)||\ =\ \left| \sqrt{2K\left( \tfrac{\pi }{4},\pi ,\alpha \rightarrow 0\right) }\right| \cdot ||\tilde{M}(\tfrac{\pi }{4},\pi ,\alpha \rightarrow 0)||. \end{aligned}$$

Now it can be easily verified that $\left| \sqrt{2K\left( \tfrac{\pi }{4},\pi ,\alpha \rightarrow 0\right) }\right| \rightarrow \infty$. Therefore it suffices to show that $||\tilde{M}(\tfrac{\pi }{4},\pi ,\alpha \rightarrow 0)||$ is strictly positive for any choice of the norm. For the spectral norm one has $||\tilde{M}(\tfrac{\pi }{4},\pi ,\alpha \rightarrow 0)||=2$. However, all matrix norms for finite dimensional matrices of a fixed dimension are equivalent, which means that for any two norms $||\cdot ||_X$ and $||\cdot ||_Y$ there exist two *positive* numbers $x,x'$ such that for any matrix *A* one has $x||A||_X\le ||A||_Y\le x'||A||_X$. From this property it follows that $||\tilde{M}(\tfrac{\pi }{4},\pi ,\alpha \rightarrow 0)||$ must be strictly positive for any choice of the matrix norm, which implies $||M(\tfrac{\pi }{4},\pi ,\alpha \rightarrow 0)||\rightarrow \infty$ and therefore the success probability for filtering the states in the neighbourhood of $\chi =\tfrac{\pi }{4}$ and $\alpha =0$, for any implementation of the SLOCC operation in Eq. (24), is arbitrarily close to zero.

This shows that our protocol overcomes the difficulties of state generation via SLOCC filtering operations, since in the latter: (i) the filtering probability can vanish, (ii) we are confined within one of the six entanglement classes depending on the initial state of the filtering. Both restrictions do not apply to our protocol, in which the success probability is constant for any state and we can reach an arbitrary state from the same trivial initial state.

## Data Availability

All data generated or analysed during this study are included in this published article.

## References

[1] Bell J. S. *Speakable and Unspeakable in Quantum Mechanics* (Cambridge University Press, 1987).

[2] Brunner N, Cavalcanti D, Pironio S, Scarani V, Wehner S (2014). Bell nonlocality. Rev. Mod. Phys..

[3] Nielsen MA, Chuang IL (2000). Quantum Computation and Quantum Information.

[4] Horodecki R, Horodecki P, Horodecki M, Horodecki K (2009). Quantum entanglement. Rev. Mod. Phys..

[5] Reck M, Zeilinger A, Bernstein HJ, Bertani P (1994). Experimental realization of any discrete unitary operator. Phys. Rev. Lett..

[6] Migdał P, Rodríguez-Laguna J, Oszmaniec M, Lewenstein M (2014). Multiphoton states related via linear optics. Phys. Rev. A.

[7] Pan J-W, Chen Z-B, Lu C-Y, Weinfurter H, Zeilinger A, Żukowski M (2012). Multiphoton entanglement and interferometry. Rev. Mod. Phys..

[8] Krenn M, Malik M, Fickler R, Lapkiewicz R, Zeilinger A (2016). Automated search for new quantum experiments. Phys. Rev. Lett..

[9] Erhard M, Krenn M, Zeilinger A (2020). Advances in high-dimensional quantum entanglement. Nat. Rev. Phys..

[10] Wang J, Sciarrino F, Laing A, Thompson MG (2020). Integrated photonic quantum technologies. Nat. Photonics.

[11] Kent A, Linden N, Massar S (1999). Optimal entanglement enhancement for mixed states. Phys. Rev. Lett..

[12] Dür W, Vidal G, Cirac JI (2000). Three qubits can be entangled in two inequivalent ways. Phys. Rev. A.

[13] Verstraete F, Dehaene J, DeMoor B (2001). Local filtering operations on two qubits. Phys. Rev. A.

[14] Blasiak P, Markiewicz M (2019). Entangling three qubits without ever touching. Sci. Rep..

[15] Yurke B, Stoler D (1992). Einstein–Podolsky–Rosen effects from independent particle sources. Phys. Rev. Lett..

[16] Yurke B, Stoler D (1992). Bell’s-inequality experiments using independent-particle sources. Phys. Rev. A.

[17] Neder I, Ofek N, Chung Y, Heiblum M, Mahalu D, Umansky V (2007). Interference between two indistinguishable electrons from independent sources. Nature.

[18] Bose S, Home D (2013). Duality in entanglement enabling a test of quantum indistinguishability unaffected by interactions. Phys. Rev. Lett..

[19] Blasiak P, Borsuk E, Markiewicz M, Kim Y-S (2021). Efficient linear-optical generation of a multipartite W state. Phys. Rev. A.

[20] Acín A, Andrianov A, Costa L, Jané E, Latorre JI, Tarrach R (2000). Generalized schmidt decomposition and classification of three-quantum-bit states. Phys. Rev. Lett..

[21] Carteret HA, Higuchi A, Sudbery A (2000). Multipartite generalization of the Schmidt decomposition. J. Math. Phys..

[22] Lo Franco R, Compagno G (2016). “Quantum entanglement of identical particles by standard information-theoretic notions”. Sci. Rep..

[23] Lo Franco R, Compagno G (2018). Indistinguishability of elementary systems as a resource for quantum information processing. Phys. Rev. Lett..

[24] Blasiak P, Borsuk E, Markiewicz M (2021). On safe post-selection for Bell tests with ideal detectors: Causal diagram approach. Quantum.

[25] Bellomo B, Lo Franco R, Compagno G (2017). N identical particles and one particle to entangle them all. Phys. Rev. A.

[26] Kim Y-S, Pramanik T, Cho Y-W, Yang M, Han S-W, Lee S-Y, Kang M-S, Moon S (2018). Informationally symmetrical Bell state preparation and measurement. Opt. Express.

[27] Kim Y-S, Cho Y-W, Lim H-T, Han S-W (2020). Efficient linear optical generation of a multipartite W state via a quantum eraser. Phys. Rev. A.

[28] Ju L, Yang M, Paunković N, Chu W-J, Cao Z-L (2019). Creating photonic GHZ and W states via quantum walk. Quantum Inf. Process..

[29] Borsuk, E. & Blasiak, P. Generation of arbitrary Dicke states in a linear optical circuit (2022) (**in preparation**).

[30] Lee D, Pramanik T, Cho Y-W, Lim H-T, Chin S, Kim Y-S (2022). Entangling three identical particles via spatial overlap. Opt. Express.

[31] Wang H, Qin J, Ding X, Chen M-C, Chen S, You X, He Y-M, Jiang X, You L, Wang Z, Schneider C, Renema JJ, Hofling S, Lu C-Y, Pan J-W (2019). Boson sampling with 20 input photons and a 60-mode interferometer in a $$10^{14}$$-dimensional Hilbert space demonstration. Phys. Rev. Lett..

[32] Tschernig K, Müller C, Smoor M, Kroh T, Wolters J, Benson O, Busch K, Perez-Leija A (2021). Direct observation of the particle exchange phase of photons. Nat. Photon..

[33] Lo Franco R (2021). Directly proving the bosonic nature of photons. Nat. Photon..

[34] Krenn M, Hochrainer A, Lahiri M, Zeilinger A (2017). Entanglement by path identity. Phys. Rev. Lett..

[35] Kysela J, Erhard M, Hochrainer A, Krenn M, Zeilinger A (2020). Path identity as a source of high-dimensional entanglement. Proc. Natl. Acad. Sci. U.S.A..

[36] Nosrati F, Castellini A, Compagno G, Lo Franco R (2020). Robust entanglement preparation against noise by controlling spatial indistinguishability. NPJ Quantum Inf..

[37] Niemietz D, Farrera P, Langenfeld S, Rempe G (2021). Nondestructive detection of photonic qubits. Nature.

[38] Johnson BR, Reed MD, Houck AA, Schuster DI, Bishop LS, Ginossar E, Gambetta JM, DiCarlo L, Frunzio L, Girvin SM, Schoelkopf RJ (2010). Quantum non-demolition detection of single microwave photons in a circuit. Nat. Phys..

[39] Munro WJ, Nemoto K, Beausoleil RG, Spiller TP (2005). High-efficiency quantum-nondemolition single-photon-number-resolving detector. Phys. Rev. A.

[40] Chin S, Kim Y-S, Lee S (2021). Graph picture of linear quantum networks and entanglement. Quantum.

[41] Avron JE, Kenneth O (2009). Entanglement and the geometry of two qubits. Ann. Phys..

